# Oral Nutritional Supplements and Enteral Nutrition in Patients with Gastrointestinal Surgery

**DOI:** 10.3390/nu13082655

**Published:** 2021-07-30

**Authors:** Maria Wobith, Arved Weimann

**Affiliations:** Department of General, Visceral, and Oncological Surgery, Klinikum St. Georg gGmbH, 04129 Leipzig, Germany; maria.wobith@sanktgeorg.de

**Keywords:** enteral nutrition, oral nutritional supplements, perioperative nutrition, malnutrition, sarcopenia, gastrointestinal surgery

## Abstract

Nowadays, patients undergoing gastrointestinal surgery are following perioperative treatment in enhanced recovery after surgery (ERAS) protocols. Although oral feeding is supposed not to be stopped perioperatively with respect to ERAS, malnourished patients and inadequate calorie intake are common. Malnutrition, even in overweight or obese patients, is often underestimated. Patients at metabolic risk have to be identified early to confirm the indication for nutritional therapy. The monitoring of nutritional status postoperatively has to be considered in the hospital and after discharge, especially after surgery in the upper gastrointestinal tract, as normal oral food intake is decreased for several months. The article gives an overview of the current concepts of perioperative enteral nutrition in patients undergoing gastrointestinal surgery.

## 1. Introduction

In an overweight or even obese society, obvious malnutrition seems to be a rare phenomenon even in patients undergoing abdominal surgery. Furthermore, nutritional therapy, either the enteral or even the parenteral route, seems to be very traditional in the era of enhanced recovery after surgery (ERAS) protocols. Nevertheless, malnutrition can be overlooked easily and has been described as a “silent epidemic” [1]. This is why screening for malnutrition and, if necessary, nutritional therapy is one eminent part of ERAS protocols [2].

While the term malnutrition means loss of body mass by an inadequate supply of energy, the term sarcopenia describes the loss of muscle mass. Malnutrition is often associated with sarcopenia if, in addition to storage fat, a relevant proportion of muscle mass is lost. On the other hand, sarcopenia can—often in geriatrics—develop without weight loss and thus without the criterion of malnutrition [3].

The presence of sarcopenia is often masked, especially in overweight or obese patients. In clinical terms, sarcopenic obesity is usually considerably underestimated. Hospital mortality occurred in 7% of 750 obese patients who were also malnourished [4]. The terminological differentiation between sarcopenic obesity and malnutrition in obesity is a matter of discussion.

In 2019, the Global Leadership Initiative on Malnutrition (GLIM) developed a new definition of malnutrition that is endorsed by all major nutritional and medical societies worldwide [5]. A distinction is made between phenotypic (weight loss, low body mass index, reduced muscle mass) and etiological criteria (reduced food intake or assimilation, inflammation/disease burden). For diagnosing malnutrition, a two-step procedure is recommended: In the case of positive nutritional screening, at least one phenotypic and one etiological criterion must be met.

The prognostic influence of the nutritional status on the occurrence of postoperative complications and the length of hospital stay has been shown many times retrospectively and prospectively [6,7,8], resulting in evidence-based guidelines on nutritional therapy for the surgical patient: European Society for Clinical Nutrition and Metabolism (ESPEN) 2017 [7] and Short ESPEN Practice guideline 2021 [9].

The ESPEN surgical guidelines are in line with the aim of early oral feeding in ERAS [7]:

- Integration of nutrition and nutritional status in the overall management
- Avoiding prolonged periods of sobriety preoperatively
- Start nutritional therapy as soon as metabolic risk is apparent
- Metabolic monitoring of blood sugar levels
- Reduction of factors that trigger stress and catabolism or impair gastrointestinal motility and function
- Early mobilization to stimulate protein synthesis and to maintain muscle function

It is evident that in most patients, oral/enteral feeding can be started just a few hours after abdominal surgery. This also applies to anastomoses in the upper gastrointestinal tract [6,7]. Oral food intake as early as possible is a key intervention of the ERAS program [10]. In the case of inadequate coverage of the calorie and protein requirement, perioperative nutrition therapy strives for supplementing the primarily oral diet by oral nutritional supplements (ONS) and enteral nutrition.

Recent evidence comes from a meta-analysis of 56 randomized controlled studies with 6370 patients undergoing surgery for gastrointestinal cancer. Benefits of supplementation with a glucose drink, increased protein intake, and immunonutrition were shown to reduce postoperative complications (RR 0.74, 95% CI 0.69–0.80), postoperative infections (RR 0.71, 95% CI 0.64–0.79), and non-infectious complications (RR 0.79, 95% CI 0.71–0.87) with a reduction in length of hospital stay of 1.58 days (95% CI −1.83 to −1.32). Nevertheless, there is considerable heterogeneity of the included studies (I = 89%) [11].

## 2. Who Will Benefit from Perioperative Nutritional Supplementation?

### Screening and Assessment of Nutritional Status

The functional status, which is pivotal for enhanced postoperative recovery, is largely determined by muscle mass. From a metabolic point of view, the combination of impaired functionality and nutritional status is crucial for postoperative mobilization and lung function. Sarcopenic patients have a significantly increased risk for severe (*p* < 0.0001) and the total number of postoperative complications (*p* = 0.001) [12]. This is why sarcopenia may be considered to be “forcing a vicious circle” [13]. The prevalence of sarcopenia in cancer patients with chemotherapy can be up to 83% [14].

Therefore, the identification of risk patients is essential preoperatively. In elderly people in particular, this should include functionality and nutritional status in a “complex geriatric assessment” [15,16]. As screening for malnutrition, the ESPEN nutritional risk score (NRS), according to Kondrup [17], has been well validated for surgical patients.

While in surgical patients, “severe nutritional risk” including NRS score ≥3, may be achieved in a 71-year-old patient without any weight loss or diminished food intake scheduled for surgery of colorectal cancer, it has been demonstrated impressively in medical patients that appropriate nutritional therapy in patients with an NRS score ≥3, treatment may already favor this outcome [18].

Current data from an observational study on the implementation of the ERAS program in hospitals in the Canadian province of Alberta revealed a positive NRS as a predictor of low patient compliance in the ERAS program (<70%: OR 2.77; 95% CI 2.11–3.64; *p* < 0.001) and a trend towards longer hospital stay >5 days (OR 1.40; 95% CI 1.00–1.96; *p* = 0.052). An ERAS compliance of <70% was associated with an increased rate of postoperative complications (OR 2.69; 95% CI 2.23–3.24; *p* < 0.001) [19].

When patients are screened at risk for malnutrition, body composition should be examined for establishing GLIM criteria [5]. In addition to more “traditional” tools such as bioelectrical impedance analysis (BIA) and dual X-ray absorptiometry (DEXA), the use of routine computer tomography (CT) in cross-section L3 has been introduced. From the section derived muscle and fat mass, conclusions can be drawn for the whole body with good correlation. The skeletal muscle index at level L3 can be used as a surrogate parameter for the presence of sarcopenia [13]. During the past few years, the prognostic impact of the CT-based measurement of the body composition with the determination of the muscle mass and the radiodensity of the muscle has been shown in numerous studies, especially for oncological patients with surgical therapy [20,21,22], as shown in Table 1.

## 3. Preoperative Nutritional Therapy

**Figure 1:**
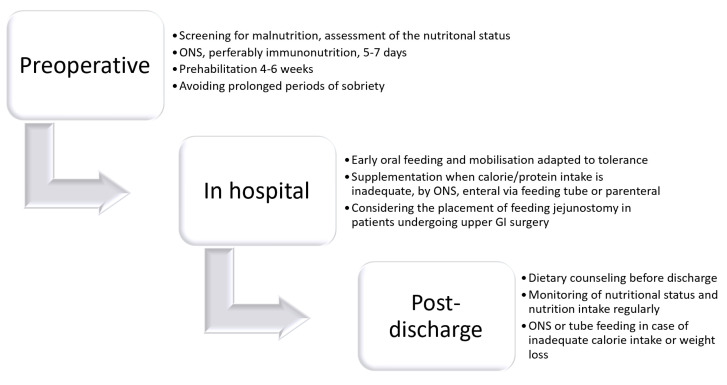
Pathway for nutritional care in patients undergoing gastrointestinal surgery.

The indication for nutritional therapy should be based on nutritional screening and by the diagnosis of malnutrition. Depending on the degree of malnutrition, the underlying disease, and the expected period of inadequately low calorie take, the decision on the duration and route of nutritional therapy has to be made (Figure 1). In case of neoadjuvant treatment with the indication for major abdominal surgery, any weight loss and deterioration of the nutritional status should be prevented.

The current ESPEN guideline [7] gives a “Good Clinical Practice (GCP)” recommendation:

“Perioperative nutritional support therapy is indicated in patients with malnutrition and those at nutritional risk. Perioperative nutritional therapy should also be initiated, if it is anticipated that the patient will be unable to eat for more than five days perioperatively. It is also indicated in patients expected to have low oral intake and who cannot maintain above 50% of recommended intake for more than seven days”.

This is in line with the updated ERAS guideline for patients undergoing elective colorectal surgery, which recommends preoperative screening for malnutrition and, if possible, oral nutrition therapy for at least 7–10 days in the case of metabolic risk or manifest deficits [2].

## 4. Prehabilitation

Current “pre-rehabilitation” conditioning concepts include a period of pre-inpatient 4–6 weeks that goes beyond the previously usual inpatient period of 10–14 days. The approach is frequently trimodal: physiotherapy, nutrition therapy, and psychological coaching in order to reduce anxiety and perioperative stress [36,37,38].

The primary goal is to make the patient “fit for ERAS” and at least to prevent further weight loss. The benefit of perioperative administration of oral nutritional supplements (ONS) has been clearly demonstrated for surgical patients with a reduction in complications and resulting in economic savings [39]. In a systematic review and meta-analysis of nine controlled studies compiled under the aspect of prehabilitation (six exclusively nutritional intervention, three multimodal), a significant reduction in the length of hospital stay by two days was found for a nutritional intervention over at least seven days, with supplementation being continued postoperatively [40].

Currently, however, there are no specific evidence-based recommendations for nutritional therapy in a prehabilitation program [36,37,38]. Eating a high-protein diet after exercise is recommended regarding the physiology of muscle protein synthesis. Since compliance is often limited for the intake of ONS [41,42], special and repeated motivation is required.

A start of prehabilitation can already be considered with the start of neoadjuvant treatment. It was shown that early nutrition support during neoadjuvant treatment in patients with esophageal cancer leads to less weight loss at 12 months postoperatively [43]. A weight loss of ≥10% results in a significantly higher mortality.

## 5. Immunological Conditioning

The stimulation of the immune system by enriching the diet with suitable immune-enhancing substrates is an interesting and controversially discussed concept as so-called “immunonutrition”.

The stimulation of anti-tumor T-cell activity has been shown in vitro for arginine. Anti-inflammatory effects can be expected from the administration of omega-3 fatty acids [44,45]. Numerous randomized clinical studies and their meta-analyses have examined the combination of arginine, omega-3 fatty acids, and ribonucleotides in an enriched oral drink supplement, as well as enterally [45]. Overall, clinical benefits have been shown in reducing the rate of infectious complications and length of hospital stay, as well as cost. This also applies to an ERAS program [46]. It has been a matter of discussion whether the exclusively preoperative administration offers advantages not only in comparison with a (hospital) diet but also in comparison with standard drinking food [7].

A recent meta-analysis of the available data from 16 randomized studies with 1387 surgical patients with gastrointestinal tumors (immunonutrition *n* = 715, controls *n* = 672) was aimed at this question. Here, the preoperative intake for 5–7 days led to a significant reduction in the incidence of infectious complications in comparison with a normal diet and with an isonitrogenic standard drinking food (OR 0.52; 95% CI 0.38–0.71, *p* < 0.0001). The heterogeneity of the data was low (I = 16%). There was a significant reduction in the length of stay in hospital compared to normal food and a tendency compared to standard drinking food (−1.57 days, 95% CI −2.48–0.66, *p* = 0.0007, I = 34%). Non-infectious complications and mortality were unaffected [47].

The results of this meta-analysis, with a focus on surgical patients with gastrointestinal cancer, show good study quality and acceptable heterogeneity: (1). Oral supplementation carried out exclusively preoperative for 5 days is effective, (2). In this context, immunonutrition is superior, at least in terms of the risk of infectious complications, compared to standard oral nutritional supplements. Focusing on patients undergoing esophagectomy, another recent meta-analysis including six randomized trials of perioperative immunonutrition did not show significant benefits regarding postoperative complication rate [11]. Thus far, the ESPEN guideline recommends ONS before major operations for 5–7 days, with immunomodulating supplements being preferred [7].

## 6. Postoperative Nutrition

### Early Oral Diet

A recent meta-analysis of five randomized controlled studies showed early enteral nutrition after major emergency surgery to be correlated with reduced mortality. Regarding the optimal timing and composition, the authors claimed the need for more high-quality data [48]. In comparison with parenteral nutrition, a recent PRCT showed significant benefits regarding intestinal recovery, hospital length of stay, and immune function for patients with cholangiocarcinoma and obstructive jaundice undergoing surgery [49]. Another randomized controlled trial in patients undergoing pelvic exenteration surgery compared parenteral nutrition and trophic enteral feeding (20 mL/h) via nasogastric tube. No significant difference between the two groups was observed for time to first bowel movement. Postoperative ileus occurred significantly less in enterally fed patients in the per-protocol analysis (*p* = 0.036). In regression analysis, it was shown again that time restriction from an oral diet was significantly associated with the time to first bowel movement and the postoperative complication rate (*p* < 0.0005) [50].

Generally, and even after surgery of the lower gastrointestinal tract, oral food intake can be started within hours (ESPEN guidelines A recommendation) [7]. The diet should be adapted to the individual tolerance and the operation performed; elderly patients require special attention [7].

St. Georg recommendations for postoperative oral diet:

- Very low in fat (max. 30 g fat/day)
- Use of easily digestible, lean protein carriers
- High carbohydrate
- Easily digestible (low fiber content)

## 7. Early Oral Delivery after Esophagectomy and Gastrectomy

A recent multicenter randomized Dutch study investigated the feasibility and safety of an early oral diet after minimally invasive esophagectomy with intrathoracic anastomosis [51]. In the intervention group (*n* = 65), oral intake took place without delay, while the control group (*n* = 67) was fed only via the enteral tube for 5 days. There was no significant difference in the primary endpoint time of postoperative recovery (7 vs. 8 days) and the secondary parameters of complications with anastomotic leakage (18.5% vs. 16.4%) and pneumonia rate (24.6% vs. 34.3%).

In another retrospective study in patients after gastrectomy, a group with early oral food intake from the first day (EOF *n* = 203) was compared with a historical control group with conventional delayed oral food intake (COF *n* = 203) by means of propensity score matching. The EOF group showed an earlier onset of flatus (2.9 vs. 3.1 days, *p* = 0.013). The length of stay in the hospital was significantly shorter (8.9 ± 5.7 vs. 12.6 ± 10.2 days, *p* < 0.01). No significant differences were observed for morbidity and mortality, with the EOF group showing a lower rate of abdominal infections (3.0% vs. 7.4%, *p* = 0.044) and anastomotic leakages (1.5% vs. 4.9%, *p* = 0.048). A subgroup analysis based on age, gender, surgical method, lymph node dissection, and tumor stage also showed no risk of increased morbidity or anastomotic leakage in the EOF group. The compliance with oral nutrition was the same in both groups [52]. In a prospective study with 50 patients undergoing major abdominal surgery, the protein and calorie intake in the first postoperative week was recorded. In the majority of patients, the energy and protein intake was insufficient (82% and 90%, respectively), leading to more Clavien-Dindo III complications in patients who did not meet their protein targets [53]. In terms of nutritional medicine, it must be emphasized that an early oral diet is feasible but that the oral calorie requirement is not met over a longer period of time comprising the time after discharge, even after post-inpatient, and thus initiates weight loss. This is an argument in favor of oral/enteral supplementation via sip feed/tube feed via feeding jejunostomy (FJT) placed during surgery.

## 8. Feeding Jejunostomy

Zhuang et al. retrospectively analyzed the outcome of their patients undergoing esophageal resection with and without placing a feeding jejunostomy. The feeding jejunal tube was placed if the patient was considered to be at high risk for developing anastomotic leakage. No significant difference was observed regarding the length of hospital stay, short-term mortality, and overall survival. There was a tendency in FJT patients for the recovery of anastomotic leakage (27.2 vs. 37.4 d, *p* = 0.073). The results suggest FJT being safe and that the placement should be considered in high-risk patients [54]. A meta-analysis for the comparison of jejunostomy versus nasoenteral tube showed benefits for the jejunostomy regarding postoperative pneumonia, length of hospital stay, and dislocation of the tube [55]. Similar results were shown in a Swedish register-based study, which investigated the differences between patients undergoing esophagectomy with and without intraoperative implantation of nutritional jejunostomy. The risk of developing severe complications (≥IIIb Clavien-Dindo) was significantly decreased in jejunostomy patients with an anastomotic leak. There was no increased risk for jejunostomy-related complications [56]. These arguments are for the selective use in high-risk patients undergoing esophagectomy [57], while the preoperative identification may be a matter of debate.

## 9. Oral Food Intake after Prolonged Intensive Care Treatment

Despite best perioperative management and ERAS protocol as a plan A, serious complications occur, inducing catabolism and subsequently increasing the risk of postoperative deterioration in nutritional status [58]. This needs a plan B, including enteral and even parenteral nutrition. The ESPEN guidelines state: If the energy and nutrient requirements cannot be met by oral and enteral intake alone (<50% of caloric requirement) for more than seven days, a combination of enteral and parenteral nutrition is recommended (GPP).

From a nutritional point of view, the period after transfer from intensive care to a normal ward is very vulnerable. Spontaneous oral food intake is usually inadequate. The main causes may be inappetence and fatigue with limited cooperation. In the anabolic situation, after a longer period of severe catabolism, the undersupply of energy and protein may be a “metabolic catastrophe”, increasing the risk for delayed recovery and rehabilitation. In a small cohort study, 32 patients were followed up with their food intake measured by their energy (2000; 1650–2550 kcal/d) and protein (112; 84–129 g/d) requirements after they had been transferred from the intensive care unit. A median of 1238 (869–1813) kcal/d and 60 (35–89) g protein was consumed over 227 days. Most of the patients were fed exclusively orally (55%) or combined enterally (42%). The energy and protein intake were lower than they estimated or even measured by indirect calorimetry. It was shown that energy and protein requirements could only be achieved with a combination of oral and enteral nutrition. The supplementation of an oral diet with ONS only covered about 70% of the requirement [59].

## 10. Post-Discharge Nutrition

In particular, after resections in the upper gastrointestinal tract, sustained weight loss as a “bariatric effect” has to be expected. Dietary counseling and follow-up monitoring of the nutritional status (minimum: BMI), including documentation of the amount of oral food intake, are essential.

A systematic review of 18 studies revealed a postoperative weight loss of 5% to 12% after 6 months. More than half of the patients lost >10% of their body weight after 12 months [60]. Therefore, these patients have to be considered to be at severe metabolic risk. Koterazawa et al., additionally showed that severe weight loss 3 months after esophagectomy could not be diminished by enteral tube feeding but had a significant impact on the 5-year overall survival rate [61]. The guidelines recommend the implantation of needle-catheter-jejunostomy (FIT) during surgery. Our own results in patients with esophageal and gastric resection, including partial pancreato-duodenectomy, show a weight loss of >10% in 40% of patients after 6 months, even with consistent postoperative continuation of nutritional therapy via feeding jejunostomy [16]. Early postoperative weight decreased up to 3 months, while stabilization occurred between 4–6 months after surgery; a further decline could be prevented by continuing enteral feeding supplementation [16]. In comparison with a control group, Chen et al. demonstrated in elderly patients after esophagectomy significant benefits from home enteral nutrition for at least 8 weeks for the BMI, PG-SGA score, serum albumin, and immune parameters [62].

In a recent meta-analysis, home enteral nutrition (HERN) and oral nutritional supplements were compared in patients with upper gastrointestinal resection for malignancy [63]. A total of 15 RCTs involving 1059 patients were included. Home enteral nutrition seemed to be superior by the significant prevention of weight loss (−3.95 vs. −5.82 kg; SMD: 1.98 kg; 95% CI 1.24–2.73) and reduction of the incidence of malnutrition or latent malnutrition (RR = 0.54; *p* < 0.01). Furthermore, improved levels of albumin, hemoglobin, pre-albumin, and transferrin were observed. Subgroup analysis based on the approach of home nutrition therapy showed that weight loss in the home enteral nutrition subgroup was significantly lower than that of the control group (WMD = 2.69, *p* < 0.01). No significant difference could be observed between the ONS subgroup and the control group. The same results were found in albumin, physical function (WMD: 5.29; 95% CI 1.86–8.73), and fatigue (WMD: −8.59; 95% CI −12.61, −4.58). Furthermore, dimensions in QOL were significantly better in the HERN group.

In a randomized study, significantly less bodyweight loss was observed when ONS was administered until 12 weeks after surgery in patients with total gastrectomy, which was not seen in patients with subtotal resection [64]. In another randomized study in patients after gastrectomy, dietary counseling in combination with ONS [65] showed a significant reduction in weight loss, with a higher BMI and SMI. Fatigue and loss of appetite were also significantly lower than with diet advice alone. Limited compliance in taking sip feeds has to be always considered [17], which may be caused by loss of appetite, taste, bloating, belching, and diarrhea. A recent multicentric randomized trial of 1003 patients after gastrectomy compared the impact of ONS 400 kcal/d on weight loss 1 year after gastrectomy. In the ONS group, only 50.4% of the patients had an intake of more than 200 kcal/day of ONS (average 301 mL) and showed significantly less bodyweight loss (8.2 ± 7.2%) at 1 year compared to the control (*p* = 0.0204) [66].

## 11. Conclusions

In conclusion, in patients undergoing gastrointestinal surgery with special regard to those of the upper GI tract, nutritional monitoring and therapy may be required even after discharge from the hospital. In line with the guidelines appropriate recommendations are summarized in Table 2. Oral nutritional supplementation and/or enteral nutrition provide clinical benefits for the attenuation of weight loss. From an ethical point of view, randomized studies may be only justified comparing oral versus enteral supplementation. Individualized home nutrition therapy requires a network with the cooperation of the surgeon, general practitioner, dietitian, and home care provider. Regular monitoring of body composition and quantitative registration of oral nutrition intake is mandatory. In the future, patient reporting and dietary counseling may be improved by virtual coaching and assisted digitalized communication by chatbot [67].

As an open question, the impact of perioperative oral and enteral supplementation in the framework of prehabilitation and ERAS on long-term oncological outcomes in patients with gastrointestinal cancer has to be elucidated in the future [68]. Further data from observational and controlled trials, including patient-reported outcomes, are urgently needed.

## Figures and Tables

**Table 1:** Overview of the literature that shows CT-derived sarcopenia to be predictive for outcomes in patients undergoing gastrointestinal surgery.

Year	Author	Cancer	N	Outcome
2017	Cespedes et al. [23]	Colorectal	2470	Higher mortality
2017	Palmela et al. [24]	Gastric	48	Higher mortality
2018	Kroenke et al. [25]	Colorectal	3262	Higher mortality
2018	Martin et al. [26]	Colorectal	1139	Longer LOS
2018	Pecorelli et al. [22]	Pancreas	120	Higher mortality
2019	Xiao et al. [27]	Colorectal	3051	Higher comorbidity
2019	Herrod et al. [28]	Colorectal	169	Higher comorbidity rate
2019	Lindner et al. [29]	Pancreas	139	Higher comorbidity rate
2019	Yassaie et al. [30]	Esophagus	53	Higher mortality
2020	Martin et al. [31]	Neck, Lung, GI	1157	Higher mortality
2020	Xie et al. [32]	Colorectal	132	Higher comorbidity rate, higher mortality
2020	Gruber et al. [33]	Pancreas	133	Higher comorbidity rate, higher mortality
2021	Ishida et al. [34]	Esophagus	333	Higher mortality
2021	Argillander et al. [35]	Colorectal	233	Higher mortality

**Table 2:** Perioperative recommendations summary for patients undergoing gastrointestinal surgery.

Recommendations Summary
- Malnutrition and sarcopenia are prognostic factors, a preoperative assessment of the nutritional status is mandatory - If there is a high metabolic risk/severe malnutrition, surgery should be postponed and nutritional therapy should be administered enterally for 10–14 days if possible, and prehabilitation should be considered - Preoperative use of ONS for at least 7 days may diminish the rate of infectious complications and the length of hospital stay - In patients with gastrointestinal cancer, preoperative immunonutrition for 5–7 days may lead to a reduction in infectious complications - Early oral intake is also feasible after esophagectomy and gastrectomy - If the oral calorie intake is insufficient (<50% for more than seven days), ONS and enteral supplementation may be indicated - Metabolic risk patients should receive dietary counseling at the time of discharge and follow-up nutritional care - Follow-up on the nutritional status and nutrition therapy after discharge may be necessary especially after surgery in the upper GI as prolonged weight loss with high metabolic risk is common
